# A Bio-Inspired Authenticated Key Exchange Binding AlphaFold2 Protein Geometry to Ephemeral Elliptic-Curve Diffie–Hellman

**DOI:** 10.3390/biomimetics11090602

**Published:** 2026-08-23

**Authors:** Abdullah Alabdulatif, Shahd Alqaan, Rana Abdulaziz Alhusika, Talah Abdullah Almuhawwis, Raghad Ahmed Almujaydil

**Affiliations:** 1 Department of Computer Science, College of Computer, Qassim University, Buraydah 52571, Qassim, Saudi Arabia; rn.rn.12@hotmail.com (R.A.A.); talahalmuhawwis@gmail.com (T.A.A.); raghadamj@outlook.com (R.A.A.); 2 Department of Computer Science, College of Engineering and Information Technology, Onaizah Colleges, Unaizah 51911, Qassim, Saudi Arabia; shahd.g@oc.edu.sa

**Keywords:** authenticated key exchange, elliptic-curve cryptography, ephemeral Diffie–Hellman, forward secrecy, hash-to-curve (RFC 9380), AlphaFold2, HKDF, AES-256-GCM, ProVerif, bio-inspired cryptography

## Abstract

Authenticated key exchange has been built for decades on a small number of hard mathematical problems, and the elliptic curve discrete logarithm is one of them. The structural complexity of biological macromolecules, however, has rarely been used as a source of secret material in such protocols. In this paper, a protein-derived authenticated key exchange protocol, called PDKE-2, is proposed to address this gap. The protocol keeps elliptic curve Diffie–Hellman at its core and adds two ingredients from structural biology. Firstly, a per-session base point G* is generated by applying RFC 9380 hash-to-curve to a session mapping table. Secondly, a long-term secret shared between the two parties is derived from the SHA-256 digest of an AlphaFold2 inter-residue distance matrix. In contrast to an earlier version of this design, the two parties run ephemeral Diffie–Hellman in each session and the session key is never sent over the channel; only transcript-based confirmation tags are exchanged. Thus, the protocol provides forward secrecy and avoids a confidentiality weakness that existed in the earlier version. The security of PDKE-2 is analysed in the Real-or-Random model, where session key secrecy, forward secrecy and mutual authentication are proved under the Gap Diffie–Hellman, PRF-HKDF and EUF-CMA-HMAC assumptions. In addition, the protocol is modelled in ProVerif against a Dolev–Yao attacker, and the secrecy of the session key, the secrecy of the shared secret and the injective agreement in both directions are all confirmed. A Python reference implementation is also provided. In the implementation, key agreement and mutual authentication succeed in every session, and the complete handshake takes about 15 ms in the reference code. It should be noted that the protein layer does not increase the elliptic curve security level; it works as an authentication secret whose strength depends on the difficulty of guessing the protein sequence when this sequence is kept private.

## 1. Introduction

The protection of session keys exchanged over insecure networks is one of the main goals of modern communication systems. This goal has been achieved by public key cryptography, which can be based on several hard mathematical problems; the most widely deployed schemes rest on three of them: the integer factorisation, the discrete logarithm in a finite field, and the elliptic curve discrete logarithm (ECDLP). For more than four decades, these problems have supported widely used schemes. For instance, RSA depends on the first problem, while Diffie–Hellman and ECDH depend on the second and third problems. However, this security is not permanent. Shor’s algorithm [1] is able to break the factorisation problem and both discrete logarithm problems on a large quantum computer. For this reason, the post-quantum cryptography standardisation led by NIST was started, and the first standards were published in August 2024, namely ML-KEM (FIPS 203) [2], ML-DSA (FIPS 204) [3] and SLH-DSA (FIPS 205) [4].

Lattice-based schemes, however, bring their own practical considerations. The lattice reduction algorithms are continuously improved [5]; the implementations may leak information through side channels; and the large key sizes are not suitable for constrained devices. Therefore, a number of researchers have looked for secret material outside number theory, such as biological structures [6,7]. Protein and amino-acid material has already been used for symmetric encryption, for key generation, and for steganography [6,7,8,9,10]. In this regard, however, the three-dimensional geometry of a predicted protein fold has not been used before as a secret inside an authenticated key exchange protocol, and the present work does not claim that protein material in cryptography is new, but only that this specific use of it is new. This paper aims to address this gap.

A protein is a chain that is built from twenty amino acids, and its three-dimensional shape is determined by physical interactions that are expensive to simulate. AlphaFold2 [11] predicts this shape with an accuracy that is close to experimental methods, and one of its outputs is an inter-residue distance matrix. This matrix is a dense description of the fold. For a protein sequence that is not stored in a public database, the matrix is expensive to obtain without running the prediction. Therefore, the digest of this matrix can be used as a secret that the two parties are able to compute, because they share the sequence, but the attacker is not able to compute it, because the attacker does not have the sequence.

One important point should be clarified before the design is presented. Making a protein-derived value secret does not increase the ECDLP security level, because the elliptic curve generator is normally public, and hiding it does not change the difficulty of the logarithm problem. Therefore, the protein layer here is used for authentication only. It works as a shared secret whose strength is the difficulty of guessing the protein sequence when this sequence is private. For this reason, PDKE-2 is designed as an ephemeral and mutually authenticated ECDH, in which the protein digest plays the role of a strong pre-shared key, and no claim is made that the protein bits are added to the 128-bit level of the curve. The value of using a protein digest, rather than an arbitrary pre-shared key, is threefold. Firstly, the secret is not a short reusable string that can be copied and exfiltrated easily, but is reconstructed from a sequence whose fold is expensive to obtain without running the prediction. Secondly, the secret can be bound to a restricted sequence database, or even to a physical biological sample, which makes it harder to phish than a typed password. Thirdly, as discussed in Section 5.6, the protein secret is bound into the key schedule together with a fresh ephemeral exchange, so that a passive eavesdropper cannot test candidates offline; because an active attacker still can, the sequence must be drawn from a high-entropy space.

The main contributions of this paper are as follows. Firstly, the PDKE-2 protocol is specified as a three-message authenticated key agreement that binds AlphaFold2 fold geometry to ephemeral ECDH over a hash-to-curve base point. Secondly, session key secrecy, forward secrecy and mutual authentication are proved under standard assumptions, and a reduction that was stated incorrectly in an earlier, unpublished draft of this work is corrected. Thirdly, a ProVerif model of the handshake is provided and checked. Fourthly, a Python reference implementation with a benchmark harness is released. Finally, the exact role of the biological layer is explained without exaggeration. It should be stressed that the novelty of this work is in how a high-entropy shared secret is derived from, and bound to, protein structure, and not in the cryptographic core, which is a standard pre-shared-key-authenticated ephemeral ECDH.

The rest of this paper is structured as follows. Section 2 will review the related work. Section 3 will present the preliminaries. Section 4 will describe the PDKE-2 protocol. Section 5 will provide the security analysis. Section 6 will show the implementation and the evaluation. Section 7 will discuss the properties and the limitations. Section 8 will present the conclusions and future work.

## 2. Related Work

### 2.1. Biological Substrates for Cryptography

Using biomolecules as a cryptographic resource goes back to Adleman’s DNA solution of a Hamiltonian-path instance [12], which seeded a large literature surveyed by Mahjabin et al. [13], including DNA one-time pads, keystreams drawn from nucleotide sequences, and steganography inside synthetic strands. Two limits recur here. The four-letter alphabet gives only 4^L strings for a length L, and the synthesis and sequencing of DNA need a wet laboratory. Protein-based schemes widen the alphabet to twenty (20^L strings) and, more importantly, carry spatial information that a bare sequence does not. Several protein-based constructions have therefore been proposed. The proteinoid-assembly cryptography of Mougkogiannis et al. [6] and the amino-acid encryption of Sakr et al. [7] both work from sequence-level or biochemical features, and Mawla and Khafaji [10] combine protein chains with steganography. More recently, Tirmazi et al. [9] introduced protein cryptography as a concept and proposed a symmetric scheme that is based on the biological properties of proteins. Closest to the present work, Al-Husainy et al. [8] used a three-dimensional protein structure as a key for image encryption. However, all of these schemes operate in the symmetric setting, and they draw on the amino-acid sequence, on biochemical properties, or on a general three-dimensional structure, rather than on a predicted inter-residue distance matrix. Moreover, none of the protein-based schemes discussed above provides an authenticated key exchange or a formal security proof. Therefore, what has not been done before, and what this paper addresses, is binding AlphaFold2-predicted fold geometry to an authenticated key exchange and proving the result secure.

### 2.2. Keys from Hardware and Biometrics

Physical unclonable functions [14] derive device-unique keys from manufacturing variation and remain popular for lightweight authentication [15]. Fuzzy extractors [16] bind keys to noisy biometrics such as fingerprints or iris codes. Both extract entropy from a physical source rather than a mathematical assumption, and both have real drawbacks. PUFs fall to machine-learning modelling attacks [14], and biometrics raise privacy questions under GDPR-style regimes. Moreover, neither offers the structural richness of a predicted fold, and neither has been tied to elliptic-curve key exchange. PDKE-2 is complementary in this respect, because a PUF-derived or biometric-derived salt could be included in its HKDF step for extra binding.

### 2.3. Hashing to Elliptic Curves

Before RFC 9380 [17], mapping a byte string to a curve point usually meant try-and-increment, that is, hash the input, test the x-coordinate for curve membership, and increase a counter until it works. Icart’s encoding [18] and the analysis around it made clear that such loops run in variable time and can leak through timing. Fouque and Tibouchi [19] and Brier et al. [20] developed constant-time, indifferentiable maps, of which the simplified Shallue–van de Woestijne–Ulas (SSWU) map is the one RFC 9380 standardises for P-256, with uniformity and pseudo-randomness proved in the random-oracle model, and more recent work such as SwiftEC [21] improves the efficiency further. In the present protocol, the P-256 suite of the standard is adopted without modification, and its guarantees are inherited.

### 2.4. Models for Authenticated Key Exchange

Security definitions for key exchange have accreted through a sequence of models: Bellare–Rogaway [22] gave the first game-based definition (a fresh key must look random); the eCK model [23] admitted reveal of ephemeral and long-term secrets under controlled conditions; Canetti–Krawczyk [24] added session-state reveal. In the present analysis, the Real-or-Random (RoR) formulation of Abdalla, Fouque, and Pointcheval [25] is used, because it composes cleanly when the session key is later consumed by symmetric encryption. On the construction side, the sign-and-MAC line of SIGMA [26] and HMQV [27], and its influence on TLS 1.3 [28], guide the use of transcript-based confirmation in PDKE-2. The difference is that the authentication here comes from a shared protein secret rather than from a signature key.

Relative to standard pre-shared-key channels such as TLS-PSK and IKEv2 with pre-shared keys, PDKE-2 keeps the same cryptographic core, namely a pre-shared-key-authenticated ephemeral ECDH, and differs only in the origin of the shared secret: it is reconstructed from private protein structure rather than provisioned as a static key. Unlike a password-authenticated key exchange (PAKE), PDKE-2 does not add offline-dictionary resistance (Section 5.6), so its shared secret must be high-entropy; the value it offers over an ordinary pre-shared key is therefore the hard-to-phish, reconstructable, physically bindable source of the secret, not a stronger cryptographic guarantee.

### 2.5. Comparison with Prior Bio-Inspired Schemes

Table 1 places PDKE-2 next to the closest prior bio-inspired schemes. The comparison is made along four axes that matter for a key-exchange protocol: the biological source that is used, whether the scheme is symmetric or an authenticated key exchange, whether a formal security proof is given, and whether a working implementation is provided. As the table shows, earlier schemes work in the symmetric setting and draw on the amino-acid sequence, on biochemical properties, or on a general three-dimensional structure, and none of them provides an authenticated key exchange with a formal proof. PDKE-2 differs on each of these axes.

## 3. Preliminaries

### 3.1. Elliptic Curves and the Diffie–Hellman Assumptions

The material in this section is standard background; the elements specific to PDKE-2 are the per-session base point of Section 3.2 and the protein-derived secret of Section 3.4. Over a prime field F_p, an elliptic curve in short Weierstrass form is y^2^ = x^3^ + ax + b (mod p) with 4a^3^ + 27b^2^ ≠ 0. Its points, with the point at infinity O, form an abelian group under the chord-and-tangent law; Hasse’s theorem bounds the order as ||E(F_p)| − (p + 1)| ≤ 2√p, and that order factors as *n*·h with *n* prime and h the cofactor. For P-256 (secp256r1), *n* ≈ 2^256^ and h = 1. Scalar multiplication Q = [d]P costs O(log d) group operations. The relevant hard problems are:

- **ECDLP:** given G and Q = [d]G, find d. Pollard’s rho needs about √*n* ≈ 2^128^ operations on P-256.
- **Computational DH (CDH):** given [x]G and [y]G, compute [xy]G.
- **Gap-DH:** solve CDH even with access to a decisional-DH oracle. PDKE-2’s secrecy reduces to Gap-DH, and not to ECDLP, which is a point that an earlier version got wrong.

**Definition** **1** (Gap-DH advantage)**.** *For an algorithm B with a DDH oracle O, Adv^GapDH_{E,G}(B) = Pr[B^O([x]G,[y]G) = [xy]G: x,y ← Z_n]. The Gap-DH assumption on P-256 says this is negligible for every PPT B.*

Adv^GapDH_{E,G}(B) = Pr[B^O([x]G, [y]G) = [xy]G : x,y ← Z_n](1)

### 3.2. Hash-to-Curve (RFC 9380, SSWU for P-256)

hash_to_curve maps a byte string, under a domain-separation tag DST, deterministically to a curve point. Here, the P256_XMD:SHA-256_SSWU_RO_ suite of RFC 9380 [17] is used: hash_to_field expands the input to two field elements with expand_message_xmd over SHA-256; each is sent through the simplified SWU map; the two points are added; and the cofactor is cleared (a no-op for P-256, kept for generality). The output is indistinguishable from a uniform curve point in the ROM, and the map runs in constant time. The domain-separation tag used here is DST = “PDKE2-v1-P256-protein-basepoint”. The session base point is

G* = hash_to_curve(SHA-256(M_table_bytes), DST) ∈ E(F_p).(2)

Because G* is derived from a per-session mapping table, it differs from session to session, which gives clean domain separation between sessions. However, it should be stressed what this is not: G* is public, and its per-session variability is not a secret and adds nothing to the ECDLP level. Its 16-byte seed is sent in the clear in the first message, so both parties compute the same G* without any private exchange. Thus, an agreement gap that was left implicit in the earlier design is also closed.

### 3.3. Key Derivation and Symmetric Primitives

HKDF [29] over HMAC-SHA256 provides extraction and expansion: PRK = HKDF-Extract(salt, IKM), then OKM = HKDF-Expand(PRK, info, L). Under the PRF assumption on HMAC-SHA256, and given sufficient input entropy, the output is indistinguishable from uniform. AES-256-GCM [30] supplies authenticated encryption for application data once the handshake completes (a 96-bit nonce, a 128-bit tag; forgery probability on the order of 2^−128^ per attempt). HMAC-SHA256 [31] provides the transcript confirmation tags; under EUF-CMA, an adversary forging a tag without the key succeeds with probability at most about q/2^256^ by guessing, with the hash’s birthday bound (~2^128^) as the practical ceiling.

### 3.4. AlphaFold2 Distance Matrices

For a sequence S = (s_1_,…,s_L), AlphaFold2 [11] predicts per-residue coordinates; taking each residue’s Cα as its point, the inter-residue distance matrix D ∈ R^{L × L} has entries D[i,j] = ‖c_i − c_j‖_2_ in Angstroms. D is symmetric with zero diagonal, and its off-diagonal entries run from a few Angstroms for neighbours up to the fold’s overall extent. On the CASP14 blind set AlphaFold2 reached a median GDT_TS of 92.4 [32], and the AlphaFold Protein Structure Database [33] now holds predictions for over 214 million sequences, retrievable without local GPU inference; ColabFold [34,35] makes on-demand prediction widely accessible. The matrix D is serialised row-major to float32 bytes, and the following digest is computed:

k_bio = SHA-256(row_major_float32_bytes(D)) ∈ {0,1}^256^. (3)

**Figure 1:**
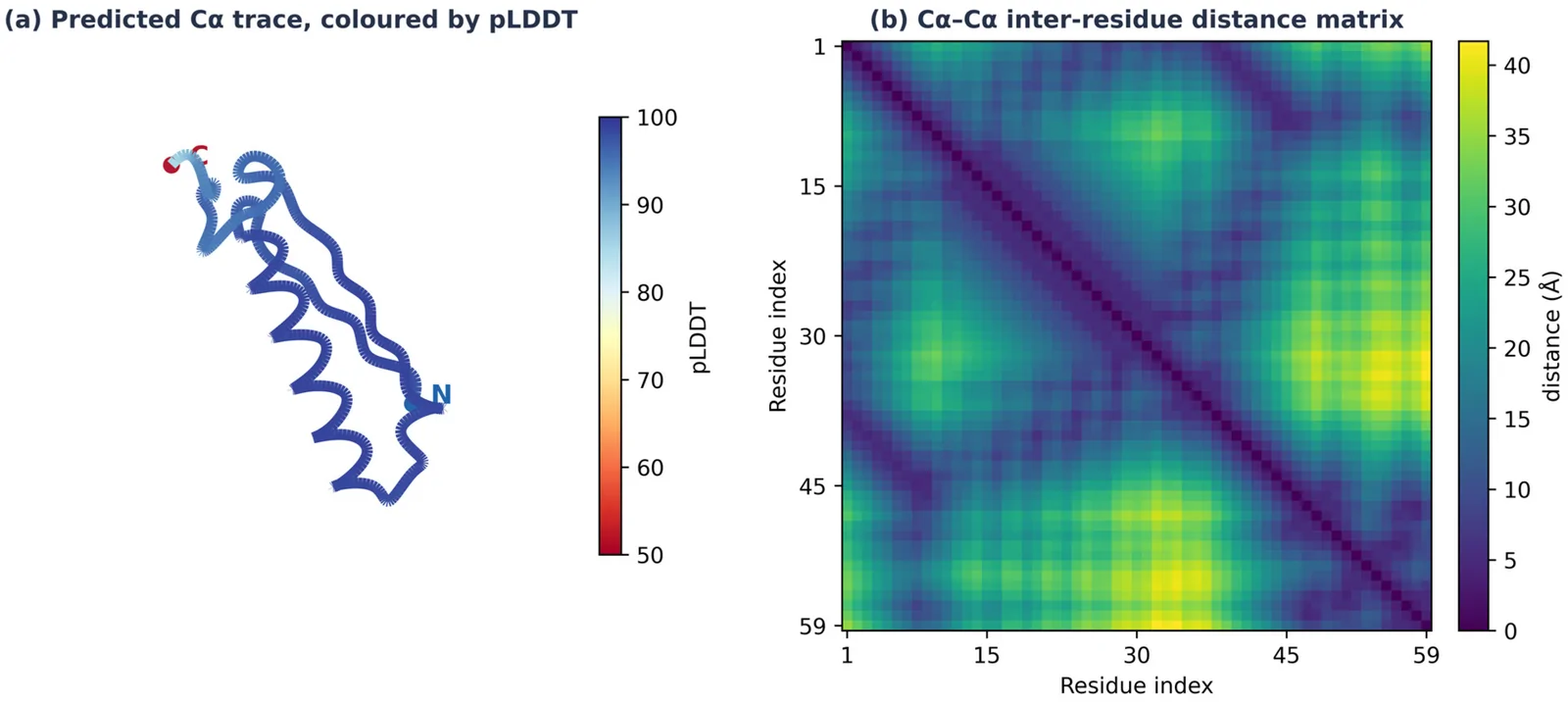
The 59-residue protein used in the evaluation (top_ranked AlphaFold2 model, mean pLDDT ≈ 97). Panel (**a**) shows the predicted Cα trace coloured by per-residue confidence (pLDDT), with the flexible C-terminus at lower confidence; panel (**b**) is the Cα–Cα distance matrix from which k_bio is derived, where darker entries mark spatially close residue pairs and the symmetric block structure reflects the compact regions of the fold.

This digest is the long-term secret *shared* between the two parties. For both parties to obtain the same k_bio, the prediction must be run in a deterministic way (fixed model, fixed seed, no dropout), and this reproducibility requirement is discussed further in Section 7. Figure 1 shows the protein that is used throughout the evaluation. Panel (a) gives the predicted Cα backbone of the AlphaFold2 model, and panel (b) gives the corresponding Cα–Cα distance matrix, which is the dense array from which k_bio is derived. The matrix is symmetric with a zero diagonal, and its off-diagonal blocks reflect the compact regions of the fold, so that spatially close residue pairs appear as dark entries and distant pairs as bright entries. It is this matrix, and not the backbone drawing, that carries the structural information used by the protocol.

## 4. The PDKE-2 Protocol

### 4.1. Parties, Secrets, and Threat Model

**Figure 2:**
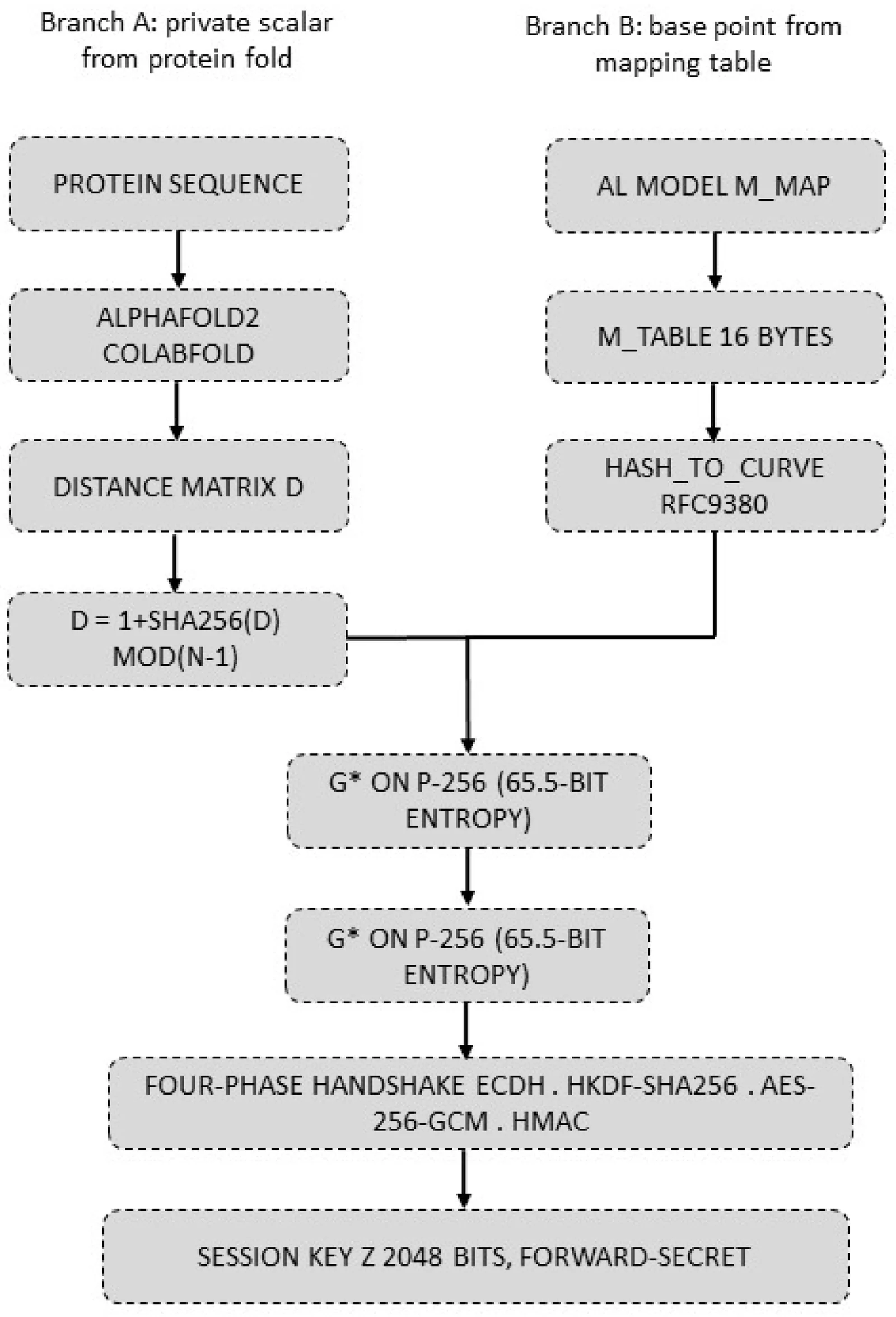
PDKE_2 architecture. The arrows indicate the direction of derivation (data flow) from the inputs to the session key, and the asterisk in G* denotes the per-session base point, which is defined in the main text.

Two parties, Alice (A) and Bob (B), share a private protein sequence Seq and can each run (or retrieve) its deterministic AlphaFold2 prediction, giving both the same k_bio of (3). Both have a CSPRNG and standard libraries for P-256, AES-256-GCM, HMAC-SHA256, HKDF, and RFC 9380 hash-to-curve. The adversary M controls the network entirely and is able to intercept, delay, replay, modify and inject messages. However, M cannot break AES-256-GCM, invert SHA-256, solve Gap-DH on P-256, distinguish HKDF output from random, forge HMAC-SHA256 without the key, or guess k_bio when Seq is private. Figure 2 gives the two ingredients and how they meet. These are computational assumptions on the abilities of M, not access-control restrictions: M is free to attempt any of them, but is assumed unable to succeed with non-negligible probability.

### 4.2. Per-Session Setup

Alice picks a fresh 16-byte seed, from which a mapping table M_table (a bijection from the sixteen 4-bit strings onto sixteen of the twenty amino acids) is derived and serialised; G* follows from (2). The seed is public and is carried in the first message, so Bob reconstructs the identical G*. Each party then draws a per-session ephemeral scalar and forms its ephemeral public point:

e_A ← Z_n*, E_A = [e_A] G*; e_B ← Z_n*, E_B = [e_B] G*.(4)

The ephemeral scalars, and not any protein-derived value, are what make the exchange forward-secret. The secret k_bio never multiplies a curve point, and it enters only through the key-derivation step, where it provides authentication. Concretely, a SHA-256 key stream keyed by the public seed drives a Fisher-Yates shuffle of the twenty amino acids; the first sixteen are assigned, in order, to the sixteen 4-bit strings, and the resulting sixteen-letter string is the serialised M_table. Both parties derive the identical table from the public seed, and G* follows from (2).

### 4.3. Three-Message Handshake

**Figure 3:**
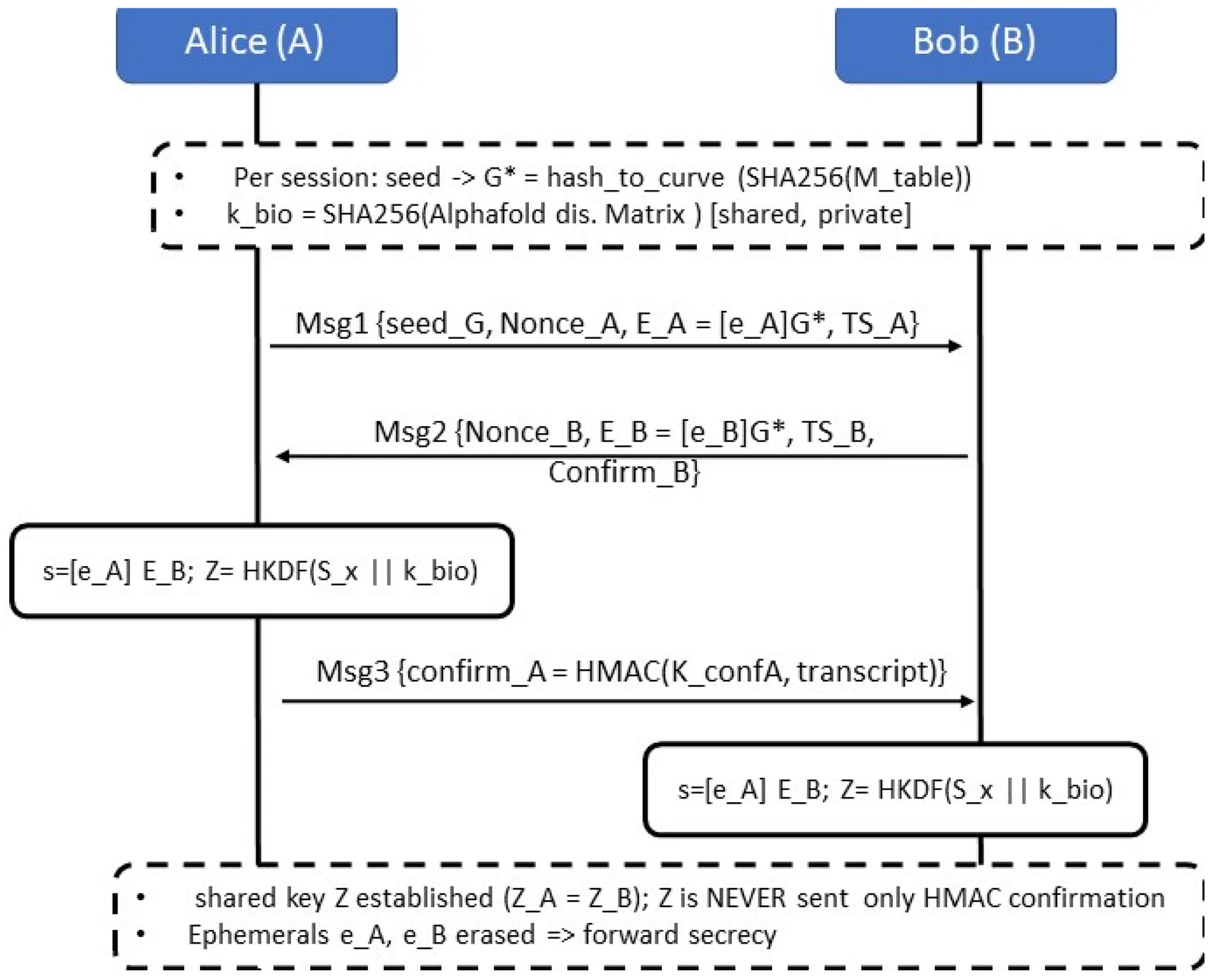
PDKE-2 handshake. The arrows indicate the message flow of the handshake (from Alice to Bob and back), and the asterisk in G* denotes the per-session base point, defined in the main text.

All three messages travel in the clear: nothing secret is transmitted, so no message needs encryption for the handshake itself. Figure 3 shows the flow.

Phase 1: Alice → Bob (msg1)

Alice draws Nonce_A (96 bits) and timestamp TS_A and sends msg1 = {seed_G, Nonce_A, E_A, TS_A} in plaintext (Equation (5)).

msg1 = {seed_G, Nonce_A, E_A, TS_A}(5)

Phase 2: Bob → Alice (msg2)

Bob checks |TS_now − TS_A| ≤ 30 s, reconstructs G* from seed_G, draws e_B, Nonce_B, TS_B, forms E_B, and computes the ephemeral shared point S = [e_B]E_A with x-coordinate S_x. The 30 s window follows common practice for timestamp-based replay protection (as in Kerberos), trading a small clock-skew allowance against the replay horizon, and is a configurable deployment parameter. He derives the session key and confirmation sub-keys by

Z = HKDF(IKM = S_x ‖ k_bio, salt = Nonce_A ‖ Nonce_B, info = “PDKE2-session-key”),(6)

(K_confA, K_confB, K_app) = HKDF-Expand(Z, labels),(7)

computes confirm_B = HMAC(K_confB, τ) over the transcript τ = H(seed_G ‖ Nonce_A ‖ E_A ‖ TS_A ‖ Nonce_B ‖ E_B ‖ TS_B), which binds both timestamps so that they are authenticated by the confirmation tags and cannot be altered by the adversary, and sends msg2 = {Nonce_B, E_B, TS_B, confirm_B} in plaintext (Equation (8)).

msg2 = {Nonce_B, E_B, TS_B, confirm_B}(8)

Phase 3: Alice → Bob (msg3)

Alice validates TS_B, computes S = [e_A]E_B (so S_x matches Bob’s by commutativity), derives Z and the sub-keys by (6) and (7), and checks confirm_B. Only if it verifies does she accept; she then returns confirm_A = HMAC(K_confA, τ) as msg3 = {confirm_A} (Equation (9)).

msg3 = {confirm_A = HMAC(K_confA, τ)}(9)

Phase 4: Bob Accepts

Bob checks confirm_A; if it verifies, he accepts. Both parties now hold the same Z and derive application traffic keys from K_app; they erase e_A, e_B, and S. Correctness follows from commutativity of scalar multiplication:

[e_A] E_B = [e_A e_B] G* = [e_B e_A] G* = [e_B] E_A ⇒ S_x^A = S_x^B ⇒ Z_A = Z_B.(10)

Because Z is fed by both S_x and k_bio, two parties reach the same Z only if they share k_bio; an attacker who does not know k_bio cannot derive Z even after solving the ephemeral DH, and an attacker who cannot solve the DH cannot derive Z even if k_bio later leaks. Application records are then protected with AES-256-GCM under K_app using a fresh 96-bit nonce per record (a per-session counter is sufficient because K_app is unique to the session); the parties rekey before the counter could repeat a nonce, and K_app is discarded when the connection closes. Table 2 summarises the steps.

## 5. Security Analysis

### 5.1. Model

The analysis is carried out in the Real-or-Random AKE model [25]. In this model, a PPT adversary A controls the network through Send queries, obtains session keys through Reveal queries, obtains the long-term secret (here k_bio) through Corrupt queries, and issues one Test query that returns either the real key or a random key. The adversary A wins if it distinguishes between the two cases for a fresh and unexposed session. The advantage of A is defined as Adv^{AKE}_{PDKE-2}(A) = |2·Pr[A wins] − 1|.

### 5.2. Theorem 1: Session-Key Secrecy

**Theorem** **1.** *For any PPT A making at most q_s Send and q_n distinct-nonce sessions,*

Adv^{AKE}_{PDKE-2}(A) ≤ 2·Adv^{GapDH}_{P-256}(B_1_) + 2·Adv^{PRF}_{HKDF}(B_2_) + 2·Adv^{EUF-CMA}_{HMAC}(B_3_) + q_n^2^/2^97^. (11)

**Proof** (sketch)**.** The proof uses a sequence of games G_0_…G_4_. G_0_ is the real experiment. In G_1_, the ephemeral DH value S = [e_A e_B]G* is replaced by a random group element. Any distinguisher for this game gives a Gap-DH solver B_1_, where the DDH oracle answers the consistency checks that the simulation needs, so |Pr[G_0_] − Pr[G_1_]| ≤ Adv^{GapDH}(B_1_). It should be noted that this is the step that the earlier version attributed incorrectly to ECDLP, because distinguishing a DH value from a random value is a decisional or gap problem, not the plain logarithm. In G_2_, the HKDF output Z and its expansions are replaced by random values, and the difference is bounded by Adv^{PRF}_{HKDF}(B_2_). In G_3_, the HMAC confirmations are replaced by random strings, and the difference is bounded by Adv^{EUF-CMA}_{HMAC}(B_3_). In G_4_, nonce collisions across q_n sessions are excluded up to the birthday bound q_n^2^/2^97^ for 96-bit nonces. After that, the key is uniform and the advantage of A is zero. Collecting the hops with the RoR factor of two gives (11).□

### 5.3. Theorem 2: Forward Secrecy

**Theorem** **2.** *If e_A, e_B, and S are erased at session end, then later disclosure of k_bio does not compromise the past session key Z, which remains indistinguishable from random under Gap-DH.*

**Proof** (sketch)**.** Z depends on S_x and k_bio only. After erasure, recovering S_x from the transcript (which carries only E_A, E_B) is exactly Gap-DH on P-256; k_bio does not help, because it never multiplies a point and the DH value is independent of it. Hence past sessions survive disclosure of the long-term secret. This is a genuine improvement over the earlier static-scalar design, whose “forward secrecy” was only conditional on the sequence staying private; the ephemeral DH here removes that condition.□

For contrast, the limitation of the earlier design is stated plainly. With static protein-derived scalars, there is no per-session ephemeral value, so the compromise of the long-term secret exposes past sessions. In PDKE-2, this problem is removed by construction.

### 5.4. Theorem 3: Mutual Authentication

**Theorem** **3.** *Under EUF-CMA-HMAC and PRF-HKDF, no PPT adversary that does not know k_bio can make either party accept, except with negligible probability.*

**Proof** (sketch)**.** K_confA and K_confB are HKDF expansions of Z, and Z requires k_bio. An adversary ignorant of k_bio gets, by Theorem 1, a Z indistinguishable from random, hence confirmation keys indistinguishable from random; producing a valid HMAC over the fresh transcript τ then contradicts EUF-CMA. Because τ binds both nonces and both ephemeral points, a verifying confirm_A/confirm_B also fixes the peer’s contribution, giving injective agreement in each direction. Note the correction over the earlier design, where confirmation was keyed on values that were themselves transmitted; here the confirmation keys are never on the wire.□

### 5.5. Symbolic Verification (ProVerif)

**Figure 4:**
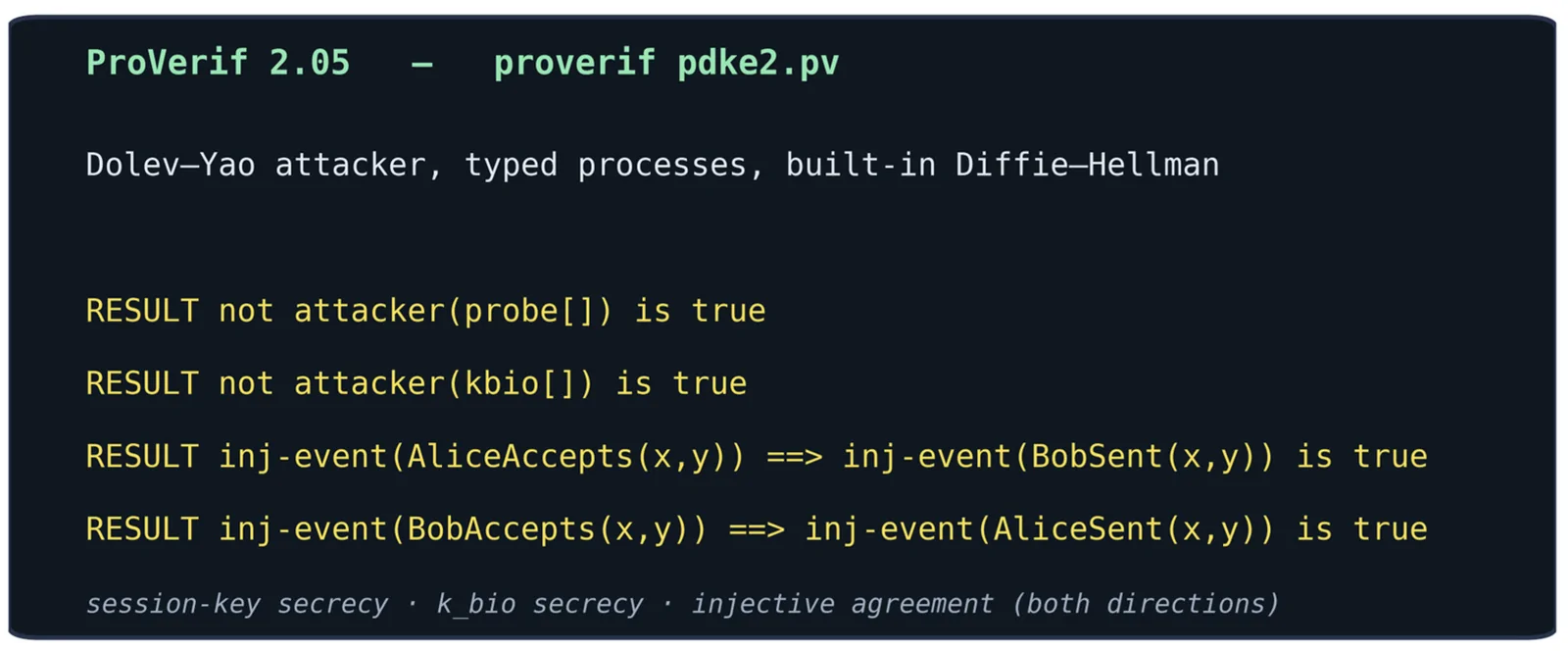
ProVerif check for PDKE-2.

The handshake is modelled in ProVerif [36] (typed processes over a public Dolev–Yao channel, ideal primitives, built-in Diffie–Hellman) in the file pdke2.pv that accompanies this paper. Secrecy of the session key is tested indirectly: Alice seals a fresh private name under a Z-derived key, and the attacker is asked to recover it. Four queries are posed: the secrecy of that value, the secrecy of the shared protein secret k_bio, and the injective agreement in each direction (Figure 4). Running proverif pdke2.pv under ProVerif 2.05 returns true for all four: not attacker(probe) is true, not attacker(kbio) is true, inj-event(AliceAccepts) ⇒ inj-event(BobSent) is true, and inj-event(BobAccepts) ⇒ inj-event(AliceSent) is true. The injective form of the agreement queries rules out replay, with ProVerif confirming that the accepting event is strictly preceded by the matching send. These verdicts hold within the symbolic abstraction, in which the cryptographic primitives are idealised and timestamps are omitted; the check therefore complements, rather than supplants, the computational proofs of Theorems 1–3. It should be emphasised that the secrecy of kbio proved here is a Dolev–Yao property: the model treats kbio as an atomic name and therefore does not capture the computational, entropy-dependent offline-guessing attack of Section 5.6, which is precisely why kbio is required to be high-entropy.

### 5.6. What the Biological Layer Adds, and What It Does Not

**Figure 5:**
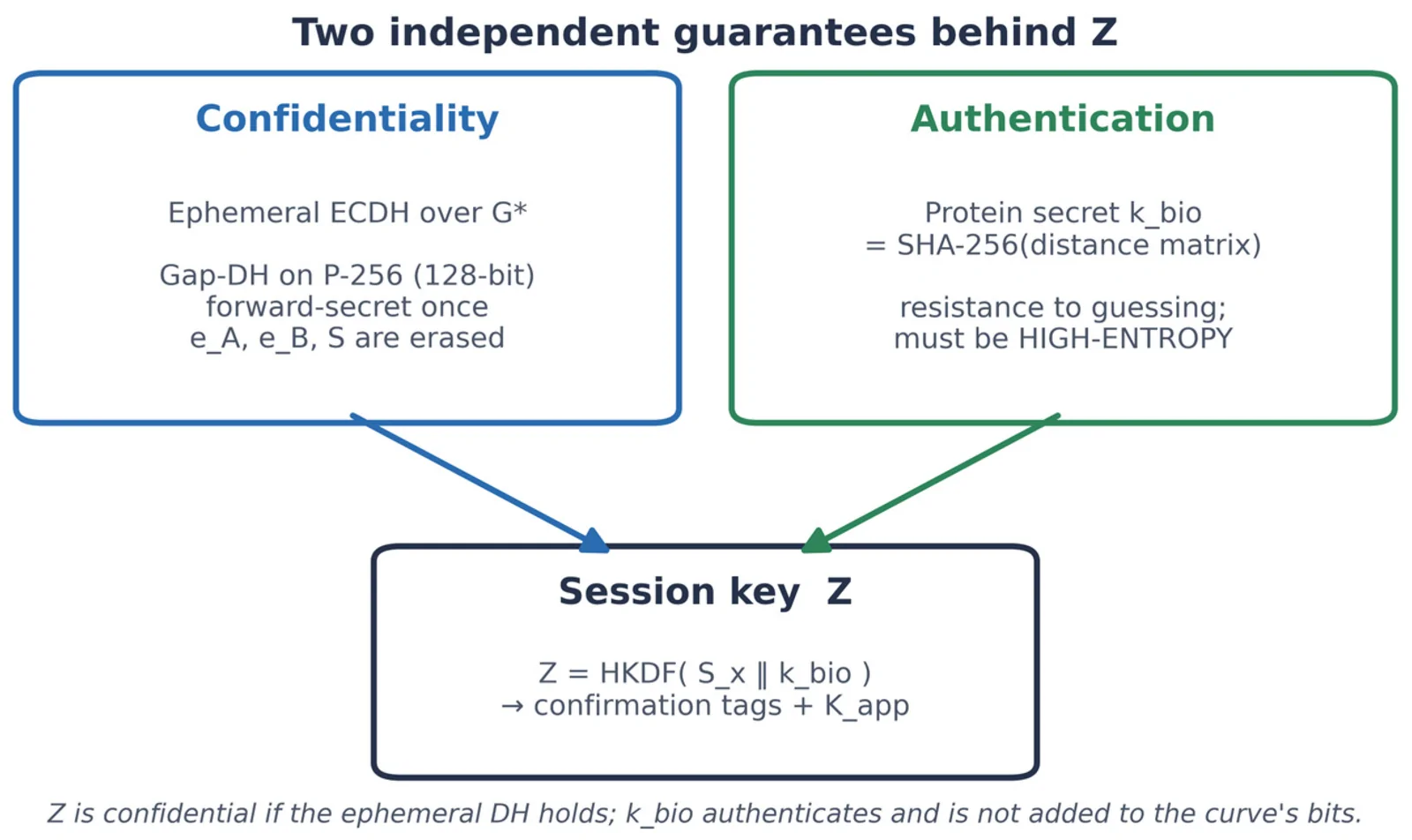
A conceptual view of the two independent guarantees behind the session key Z. the two colours distinguish the two independent guarantees behind the session key: the confidentiality branch (ephemeral ECDH, left) and the authentication branch (the protein secret k_bio, right). The asterisk in G* denotes the per-session base point.

Figure 5 states the security picture without the arithmetic sleight-of-hand of the earlier version. Confidentiality of Z rests on the ephemeral ECDH: 128-bit, Gap-DH on P-256. The protein secret k_bio sits in a different column, which is authentication. Its value is the resistance to guessing, because an attacker who wants to impersonate, or to compute Z offline, must recover the private sequence, and the difficulty of this is the min-entropy of the distribution that the sequence is drawn from, not a fixed 64 bits, and it is certainly not additive with the 128 bits of the curve. The ECDLP bits are not added to the entropy bits, because the two quantities measure different things. Thus, the clean statement is the one shown in the figure: Z is secure if either the ephemeral DH holds or k_bio is not guessed, and forward secrecy holds in any case once the ephemeral values are erased. Table 3 collects these properties together with the basis of each. Each row names the theorem or equation that establishes the property.

A limitation of the construction must be stated precisely, because it bounds the entropy that k_bio has to carry. Against a purely passive eavesdropper, testing a candidate protein secret requires the ephemeral Diffie-Hellman value S_x, which such an eavesdropper cannot obtain. An ACTIVE attacker, however, that runs a single handshake with an honest party while choosing its own ephemeral scalar learns S_x directly, and can then test candidate secrets OFFLINE against the confirmation tag it received. PDKE-2 is therefore NOT a password-authenticated key exchange and gives no protection to a low-entropy secret. Consequently k_bio must be treated as a high-entropy key: it is the full 256-bit SHA-256 digest of a distance matrix derived from a genuinely private sequence, whose min-entropy must be large enough to make offline enumeration infeasible. This requirement is made explicit here and revisited in Section 7.2. The accompanying code reproduces this offline test (pdke2.py --attack).

## 6. Implementation and Evaluation

### 6.1. Reference Implementation

A self-contained Python 3 (version 3.9 or later) reference implementation, pdke2.py, accompanies this paper. It implements the P-256 arithmetic (with scalar multiplication in Jacobian coordinates), the hash-to-curve map, and the complete three-message handshake. The cryptography library is used for HKDF and AES-GCM, and the standard library is used for SHA-256, HMAC and the CSPRNG. The SSWU map follows RFC 9380 §6.6.2 for P-256 with Z = −10. The self-test verifies that G* lies on the curve and in the prime-order subgroup, and it is recommended to validate the map against the test vectors in Appendix J.1.1 of RFC 9380 before any production use. When the file is executed, a correctness self-test is run. It confirms that G* is on the curve, that Alice and Bob derive an identical Z, that the mutual confirmation is verified, and, as a negative control, that a wrong protein secret makes the authentication fail and the keys disagree. All of these checks pass in the provided code. The reference implementation, the ProVerif model, the structure files, and the scripts used to generate the figures and the reported performance num-bers are provided in the Supplementary Materials.

### 6.2. Reproducing the Performance Numbers

Because the protocol changed relative to the earlier draft, none of the earlier timing figures are carried over; every number in Table 4 is measured afresh from the shipped code on the evaluation platform, using the harness in pdke2.py (bench_components and bench). The EC arithmetic is pure Python, so these values are conservative upper bounds, and a production build over a native P-256 backend would be markedly faster. The full-handshake figure becomes stable as the sample size increases. For instance, 100 trials give about 26 ms at the median, because the interpreter and cache warm-up dominate a small sample, whereas 1 000 and 10 000 trials both converge to a 15.33 ms median, which is the figure that is reported. Each party performs two hash-to-curve evaluations (both parties reconstruct G*), one ephemeral key generation, and one ECDH, and this accounts for the full-handshake total. AlphaFold2 inference is a one-time, out-of-band step (tens of seconds per model on a GPU via ColabFold [34,35]) and is not part of the handshake cost; in deployment, the distance matrix is pre-computed or fetched from the AFDB [33].

### 6.3. Correctness and Reproducibility

The correctness harness (check_correctness) was run over 2000 independent sessions. Alice and Bob derived an identical Z in 2000 of 2000 runs, the mutual confirmation was verified in 2000 of 2000 runs, and the negative control, a wrong protein secret, was rejected with disagreeing keys in 2000 of 2000 runs. In other words, the agreement and the mutual authentication held in every run, and no wrong-secret session was ever accepted. Because both parties must derive the same k_bio, the AlphaFold2 prediction must be run in a deterministic configuration. As a concrete illustration, the two top-ranked models of the evaluation protein differ by only about 0.13 Å on average in their Cα–Cα distances, and yet their SHA-256 digests are entirely different, because the hash amplifies any change in the input. The two parties must therefore agree on one specific model, rather than predict independently. Where this is not practical, the distance matrix should be treated as a fixed shared input (for instance, pinned in the AFDB) rather than predicted again independently. This is treated as a deployment requirement rather than as a property to be measured, and it also corrects a confusion in the earlier design, which at the same time wanted independent per-party scalars and identical agreement.

**Figure 6:**
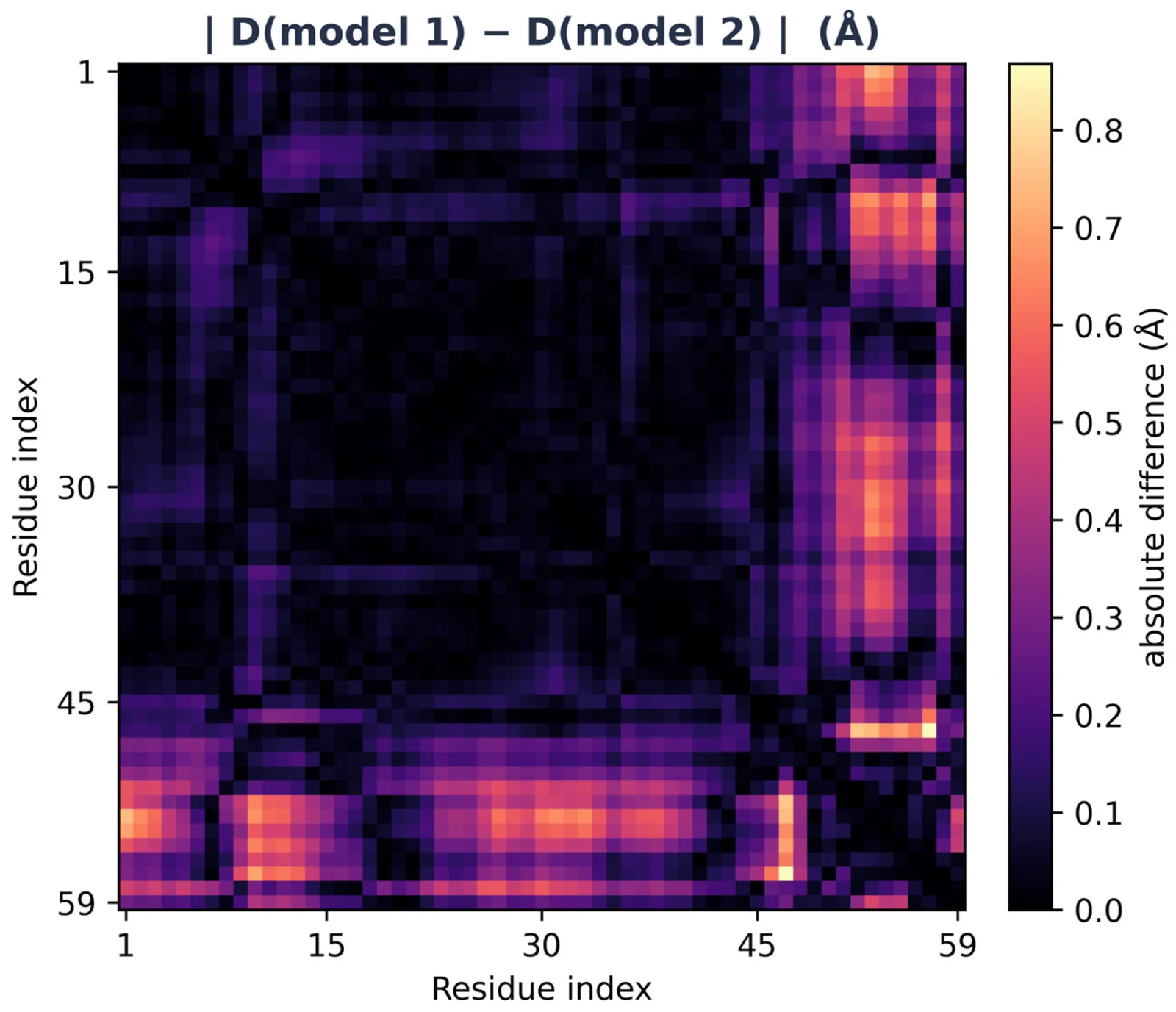
Absolute difference |D_1_ − D_2_| between the Cα–Cα distance matrices of the two top-ranked models of the evaluation protein. The differences are small everywhere and concentrate at the flexible C-terminus, yet they still change every byte of the SHA-256 digest, which is why the two parties must fix on one model.

Figure 6 makes this point visible. It shows the absolute difference between the Cα–Cα distance matrices of the two top-ranked models of the evaluation protein. The differences are small everywhere and concentrate near the flexible C-terminal residues, whose per-residue confidence is the lowest in the structure (pLDDT ≈ 84 at the last residue), while the core of the fold is almost unchanged. These small differences are nevertheless enough to change every byte of the digest. A further and stricter requirement follows from the same construction. Because k_bio is the SHA-256 of the float32 serialisation of the matrix, the two parties must agree on the model and also on the exact numerical procedure, that is, on the distance computation, on the rounding, and on the byte order of the serialisation. In practice, the distances should be rounded to a fixed precision before hashing, so that platform-dependent floating-point differences do not change k_bio.

## 7. Discussion

### 7.1. What Changed, and Why

This revision is defined by three main fixes. Firstly, no secret value is sent over the channel. In the earlier design, an initial value derived by each party was sent in the clear and was then used to “encrypt” the later messages and even to carry the session key, so a passive eavesdropper was able to open the transcript and read the key. In PDKE-2, only ephemeral public points and transcript confirmation tags are sent. Secondly, the exchange is ephemeral, so forward secrecy no longer depends on the privacy of the sequence. Thirdly, the security accounting is honest. The protein layer provides authentication, and its strength is the resistance to guessing, not extra ECDLP bits, and it is not summed with the security level of the curve.

### 7.2. Sequence Confidentiality and Entropy

An important practical point is whether the protein sequence Seq must be kept private. If Seq is public (for instance, already stored in the AFDB), then k_bio can be computed by anyone, and the protein layer gives no authentication strength. In this case, PDKE-2 reduces to an unauthenticated ephemeral ECDH, which would then be combined with a certificate or a pre-shared key as usual. Therefore, the biological layer is useful only when the sequence is private and is selected from a space that is too large to enumerate. Its resistance to guessing is the min-entropy of this selection process, which is a quantity that the designer must justify for a given deployment. For this reason, it is deliberately not fixed to a single headline number. As a concrete illustration, an engineered sequence of L residues chosen uniformly over the twenty amino acids would carry up to L·log2(20) ≈ 4.32·L bits, so about 128 bits already at L ≈ 30; realistic sequences are far from uniform, however, so it is the min-entropy of the actual selection process, not the length, that a deployment must justify, and it must exceed the level at which online guessing becomes infeasible.

### 7.3. Quantum Resistance

Like any ECC-based scheme, PDKE-2 is not post-quantum, because Shor’s algorithm breaks the ephemeral ECDH. A private protein secret does not change this: an attacker with a large quantum computer recovers S_x, and the protein layer is an authentication factor, not a post-quantum confidentiality defence. Therefore, for genuine post-quantum security, a hybrid key Z = HKDF(Z_ECDH ‖ Z_KEM) with an ML-KEM-768 [2] encapsulation is recommended. This hybrid is secure if either component is secure, and it is a small and standard extension.

### 7.4. Limitations

- **Resource cost:** AlphaFold2 needs a GPU; constrained devices must use pre-computed matrices from the AFDB [33] rather than predict at run time.
- **Reproducibility:** both parties need the same model and also the same bit-level serialisation of the distance matrix, hence a deterministic prediction, a fixed rounding precision, and a pinned reference (Section 6.3).
- **Proof model:** SHA-256 and hash-to-curve are treated in the ROM, standard for this setting; a standard-model treatment would need programmable-hash assumptions.
- **Symbolic scope:** the ProVerif model idealises primitives and abstracts timestamps; it complements, not replaces, the computational proofs.
- **Secure erasure:** forward secrecy assumes the ephemeral scalars e_A, e_B and S are securely erased at session end; in a high-level language such as the Python reference, this is best-effort only (immutable objects, garbage collection, swapping), so a production deployment must use zeroisable, locked memory in a lower-level language.
- **Role of the session base point:** G* is recomputed in every session from a public seed, which gives domain separation, but the freshness of the exchange already comes from the ephemeral scalars. The additional hash-to-curve computation is therefore a design choice that preserves the bio-inspired construction rather than a security requirement, and a deployment may replace G* with the standard P-256 generator without weakening the analysis.
- **Pair authentication only:** because k_bio is a symmetric secret shared by the two parties, the protocol authenticates membership of the pair rather than the individual identity of a party, and it does not provide non-repudiation. Where individual identities or signatures are required, an asymmetric credential should be combined with the protocol.

### 7.5. Where It Fits

PDKE-2 is suitable for settings where the two endpoints already share a confidential biological reference and need an extra and independent authentication factor on top of standard ECDH, such as closed high-assurance links, rather than open-web key exchange. In other words, it should be understood as ECDH together with a strong shared secret that has an unusual and hard-to-phish source, and not as a replacement for post-quantum KEMs.

### 7.6. When a Protein Secret Is the Right Choice

A fair question is what the protein secret adds over an ordinary pre-shared key, since the cryptographic core of PDKE-2 is a pre-shared-key-authenticated ephemeral ECDH, and the 128-bit security comes from the curve. The honest answer is that the advantage is not in the mathematics but in the origin of the secret. Three properties make a protein digest attractive as the shared secret in specific settings. Firstly, the secret is not a short string that is stored and typed, but is reconstructed from a sequence whose fold is expensive to obtain without running the prediction. The sequence can be kept out of any digital store and re-derived on demand, which reduces the surface for phishing and exfiltration that an ordinary password exposes. Secondly, the sequence can be bound to a physical or restricted asset, such as a synthesised protein sample or a confidential engineered sequence that two collaborating parties already hold as intellectual property. In that case the session key is tied to that specific asset, and compromising the channel additionally requires the confidential sequence, which is an independent factor. Thirdly, the secret is bound into the key schedule together with a fresh ephemeral exchange, which stops a passive eavesdropper from testing candidates offline; an active attacker can still do so (Section 5.6), so the sequence must be drawn from a high-entropy space.

A concrete example is two laboratories that jointly hold a proprietary engineered protein. They need a channel whose security is bound to that shared confidential design, so that an attacker who records the traffic must also obtain the design, and PDKE-2 provides exactly this binding without an extra password to manage. Where none of these properties is needed, an ordinary pre-shared key is simpler and equally secure, and PDKE-2 offers no advantage over it. The protocol is therefore aimed at settings where the biological provenance of the secret is itself the requirement, rather than at general-purpose key exchange.

## 8. Conclusions

In this paper, PDKE-2 has been presented as a protocol that binds AlphaFold2 fold geometry to an ephemeral and mutually authenticated elliptic curve Diffie–Hellman. In comparison with the earlier design, the protocol closes a confidentiality gap because no secret is sent over the channel, makes forward secrecy independent of the protein secret, corrects the main security reduction from ECDLP to Gap Diffie–Hellman, and states the role of the biological layer honestly as an authentication secret rather than as added curve strength. Proofs, a ProVerif model, and a self-checking reference implementation with a benchmark harness are provided, so that the claims, including the performance numbers, can be reproduced rather than taken on trust. In general, the results show that outputs from structural biology can be combined with standard cryptographic tools in a clean way, as long as the security accounting stays honest about what these outputs actually provide. In future work, the protocol will be extended with a post-quantum hybrid mode, and the min-entropy of realistic protein-sequence spaces will be studied in more detail.

## Figures and Tables

**Table 1:** Comparison of PDKE-2 with prior bio-inspired cryptographic schemes.

Scheme	Biological Source	Setting	Formal Proof	Authenticated Key Exchange
Adleman [12]	DNA (Hamiltonian path)	computation	No	No
DNA one-time pads (survey) [13]	DNA sequences	symmetric	No	No
Sakr et al. [7]	amino-acid sequence	symmetric	No	No
Mougkogiannis et al. [6]	proteinoid assemblies	symmetric	No	No
Tirmazi et al. [9]	protein biological properties	symmetric	No	No
Al-Husainy et al. [8]	3D protein structure	symmetric (image)	No	No
Mawla and Khafaji [10]	protein chains	symmetric + steganography	No	No
PDKE-2 (this work)	AlphaFold2 distance matrix	authenticated KE	Yes (RoR + ProVerif)	Yes

**Table 2:** PDKE-2 handshake summary. No secret value is ever transmitted.

Phase	Actor	Sends	Purpose
Setup	Both	none (seed sent in msg1)	Derive G* from public seed; both compute shared k_bio
1	Alice	seed_G, Nonce_A, E_A, TS_A	Offer ephemeral E_A
2	Bob	Nonce_B, E_B, TS_B, confirm_B	Offer E_B; confirm key knowledge
3	Alice	confirm_A	Confirm key; complete mutual auth
4	Bob	none (accept)	Verify confirm_A; erase ephemerals

The asterisk in G* denotes the per-session base point.

**Table 3:** Security properties and their basis. The biological layer is listed as an authentication secret, not as added ECDLP strength.

Property	Mechanism	Basis
Session-key secrecy	Ephemeral ECDH + HKDF	Gap-DH, PRF-HKDF, EUF-CMA-HMAC; Equation (11)
Forward secrecy	Ephemeral scalars, erased	Under Gap-DH, independent of k_bio; Theorem 2
Mutual authentication	Transcript HMAC confirmations keyed from Z (needs k_bio)	EUF-CMA-HMAC + PRF-HKDF; Theorem 3
Replay resistance	Timestamp ± 30 s + fresh nonces	Birthday bound q^2^/2^97^
Base-point separation	RFC 9380 DST + per-session seed	RFC 9380 §3.1/§7 (h = 1)
Authentication secret	k_bio = SHA-256 (dist. matrix)	Guessing-resistance of private Seq (not added to curve bits)

**Table 4:** Measured performance on the evaluation platform (Google Colab, single core; pure-Python reference in pdke2.py). Component rows are over *n* = 500; the full-handshake figure is over *n* = 10,000 (median 15.33 ms), which is treated as the representative number. A separate 100-trial run reads higher (~26 ms median) owing to interpreter and cache warm-up on a small sample. A production build over a native P-256 backend would be markedly faster; reproduce with pdke2.py. The full handshake performs two hash-to-curve evaluations and four scalar multiplications (ephemeral key generation and ECDH for each party); it is therefore not the sum of the isolated component rows, whose per-call overhead makes them read slightly higher, and all figures are local computation excluding network time.

Component	Mean ± Std (ms)	Median (ms)
hash_to_curve for G* (RFC 9380)	2.89 ± 0.38	2.91
Ephemeral keygen E = [e]G*	3.58 ± 0.99	2.98
ECDH shared point [e]E	3.02 ± 0.23	2.97
Full handshake (local computation; excludes network)	17.16 ± 4.03	15.33
Distance-matrix load	one-time, out of band	n/a

The asterisk in G* (shown in the table) denotes the per-session base point.

## Data Availability

The data supporting the conclusions of this article are provided as Supplementary Materials. These comprise the Python reference implementation, the ProVerif model, the AlphaFold2 structure files, and the scripts used to generate the figures and the reported performance numbers, so that all empirical results can be reproduced.

## Supplementary Materials

The following supporting information can be downloaded at https://www.mdpi.com/article/10.3390/biomimetics11090602/s1.

## Abbreviations

The following abbreviations are used in this manuscript:

**AEAD**	Authenticated encryption with associated data
**AES**	Advanced encryption standard
**AFDB**	AlphaFold Protein Structure Database
**AKE**	Authenticated key exchange
**CDH**	Computational Diffie–Hellman
**CSPRNG**	Cryptographically secure pseudorandom number generator
**DDH**	Decisional Diffie–Hellman
**ECDH**	Elliptic-curve Diffie–Hellman
**ECDLP**	Elliptic-curve discrete logarithm problem
**EUF****-****CMA**	Existential unforgeability under chosen-message attack
**Gap-DH**	Gap Diffie–Hellman
**GCM**	Galois/counter mode
**HKDF**	HMAC-based key derivation function
**HMAC**	Hash-based message authentication code
**KEM**	Key-encapsulation mechanism
**PRF**	Pseudorandom function
**PSK**	Pre-shared key
**ROM**	Random-oracle model
**RoR**	Real-or-random
**SSWU**	Simplified Shallue–van de Woestijne–Ulas
**TLS**	Transport layer security
